# Supplementary material for: An open-source framework for large-scale, flexible evaluation of biomedical text mining systems
Source: J Biomed Discov Collab. 2008 Jan 29;3:1. doi: 10.1186/1747-5333-3-1 (PMC2276192; doi:10.1186/1747-5333-3-1)
Supplement: Additional file 1 — Evaluation platform source code. The additional file contains the software evaluation framework discussed in this paper. The most current release can be downloaded from SourceForge.net [1]. See the accompanying README file for instructions on installation and use. Also included in the distribution is a collection of UIMA wrappers for some commonly used BioNLP tools and annotated corpora. [file 1747-5333-3-1-S1.zip › bionlp_uima_repository-1.0.1/docs/api/src-html/edu/uchsc/ccp/util/external/abner/Abner_Util.html]

```
001    package edu.uchsc.ccp.util.external.abner;
002    
003    import java.util.ArrayList;
004    import java.util.HashMap;
005    import java.util.List;
006    import java.util.regex.Matcher;
007    import java.util.regex.Pattern;
008    
009    import abner.Tagger;
010    import edu.uchsc.ccp.util.nlp.GenericTextUtils;
011    import edu.uchsc.ccp.util.nlp.annotation.AnnotationSet;
012    import edu.uchsc.ccp.util.nlp.annotation.Annotator;
013    import edu.uchsc.ccp.util.nlp.annotation.InvalidSpanException;
014    import edu.uchsc.ccp.util.nlp.annotation.Span;
015    import edu.uchsc.ccp.util.nlp.annotation.TextAnnotation;
016    import edu.uchsc.ccp.util.nlp.mention.ClassMention;
017    import edu.uchsc.ccp.util.nlp.mention.ClassMentionTypes;
018    
019    /**
020     * crap
021     * <p>
022     * This is an interface to Burr Settles' named entity recognizing application, ABNER. <br>
023     * <a href="http://pages.cs.wisc.edu/~bsettles/abner/">http://pages.cs.wisc.edu/~bsettles/abner/</a></br>
024     * <p>
025     * Currently, this interface allows the user to specify the two models that come prepackaged in the abner.jar file. By default the ABNER tokenization
026     * routine is used.
027     * <p>
028     * Extracted entities are returned as instances of <code>edu.uchsc.ccp.util.nlp.annotation.TextAnnotation</code>.
029     * <p>
030     * When the built-in BioCreative model is used, only protein annotations are returned. When the NLPBA model is used, protein, RNA, DNA, cell line, and
031     * cell type annotations are returned.
032     * 
033     * @author William A. Baumgartner Jr.
034     * 
035     */
036    public class Abner_Util {
037    
038        /* When the built-in BioCreative model is used, each protein annotation is associated with an Annotator with AnnotatorID=2 */
039        private final int BIOCREATIVE_MODEL_ANNOTATOR_ID = 2;
040    
041        /* When the built-in NLPBA model is used, each entity annotation is associated with an Annotator with AnnotatorID=42 */
042        private final int NLPBA_MODEL_ANNOTATOR_ID = 42;
043    
044        /*
045         * The startLookingForMatchesAt variable keeps track of where we are looking for entities in the original text. This is used for determining the
046         * entity spans (see below).
047         */
048        private int startLookingForMatchesAt;
049    
050        private Tagger abnerTagger = null;
051    
052        private boolean useBioCreativeModel = false;
053    
054        /**
055         * Create a new Abner_Util.
056         * 
057         * @param model
058         *            The two default ABNER models can be selected. For the BioCreative model, use <code>tagger.BIOCREATIVE</code>. For the NLPBA
059         *            model, use <code>tagger.NLPBA</code>.
060         */
061        public Abner_Util(int model) {
062            abnerTagger = new Tagger(model);
063    
064            /* turn on ABNER tokenization */
065            abnerTagger.setTokenization(true);
066    
067            if (model == Tagger.BIOCREATIVE) {
068                useBioCreativeModel = true;
069            }
070        }
071    
072        /**
073         * Retrieve the entities extracted by ABNER from text as instances of <code>edu.uchsc.ccp.util.nlp.annotation.TextAnnotation</code>.
074         * 
075         * @param text
076         *            The text to search for entities.
077         * @param documentCollectionID
078         *            This unique identifier for a document collection will be assigned to any annotations returned by this method.
079         * @param documentID
080         *            This unqiue identifier for a document will be assigned to any annotations returned by this method.
081         * @return a List of <code>edu.uchsc.ccp.util.nlp.annotation.TextAnnotation</code> instances, one per entity detected in the text.
082         */
083        public List<TextAnnotation> getEntities(String text, int documentCollectionID, String documentID) {
084            List<TextAnnotation> annotationsToReturn = new ArrayList<TextAnnotation>();
085    
086            /* Use ABNER to detect the entities */
087            String[][] entities = abnerTagger.getEntities(text);
088    
089            /* The rest of this method is devoted to determining the spans of the extracted entities, and creating TextAnnotations to return. */
090    
091            int annotationCount = 0;
092    
093            /* reset the index we will start looking for matches back to the beginning of the text */
094            startLookingForMatchesAt = 0;
095    
096            /* hashmap to keep track of annotations that have already been stored... this prevents duplicate annotations from being stored. */
097            HashMap<String, String> annotMap = new HashMap<String, String>();
098            for (int i = 0; i < entities[0].length; i++) {
099    
100                String entity = entities[0][i];
101    
102                /* find start/end indexes */
103                Span span = findSpan(entity, text);
104    
105                if (span != null) {
106                    String key = span.getSpanStart() + " " + span.getSpanEnd() + " " + entities[1][i];
107                    if (!annotMap.containsKey(key)) { /* make certain no duplicate annotations are stored */
108                        annotMap.put(key, "");
109    
110                        String mentionName = "";
111                        if (entities[1][i].equalsIgnoreCase("protein")) {
112                            mentionName = ClassMentionTypes.PROTEIN;
113                        } else if (entities[1][i].equalsIgnoreCase("dna")) {
114                            mentionName = ClassMentionTypes.DNA;
115                        } else if (entities[1][i].equalsIgnoreCase("rna")) {
116                            mentionName = ClassMentionTypes.RNA;
117                        } else if (entities[1][i].equalsIgnoreCase("cell_line")) {
118                            mentionName = ClassMentionTypes.CELL_LINE;
119                        } else if (entities[1][i].equalsIgnoreCase("cell_type")) {
120                            mentionName = ClassMentionTypes.CELL_TYPE;
121                        } else {
122                            System.err.println("***********   Unrecognized entity type: " + entities[1][i] + "  ***********************");
123                        }
124    
125                        // set the annotator
126                        Annotator annotator;
127                        if (useBioCreativeModel) {
128                            annotator = new Annotator(BIOCREATIVE_MODEL_ANNOTATOR_ID, "", "ABNER - BC", "WISC");
129                        } else {
130                            annotator = new Annotator(NLPBA_MODEL_ANNOTATOR_ID, "", "ABNER - NLPBA", "WISC");
131                        }
132    
133                        AnnotationSet annotationSet = new AnnotationSet(-1, "Default Annotation Set", "Default Annotation Set");
134    
135                        ClassMention cm = new ClassMention(mentionName);
136    
137                        String coveredText = text.substring(span.getSpanStart(), span.getSpanEnd());
138                        TextAnnotation ta = new TextAnnotation(span.getSpanStart(), span.getSpanEnd(), coveredText, annotator, annotationSet,
139                                annotationCount, documentCollectionID, documentID, -1, cm);
140    
141                        annotationsToReturn.add(ta);
142                        annotationCount++;
143                    } else {
144                        // key already present, so don't do anything
145                    }
146                } else {
147                    error("Null span, Could not find: \"" + entity + "\" in documentText: \"" + text + "\"");
148                }
149            }
150    
151            if (annotationCount != entities[0].length) {
152                error(entities[0].length + " annotations should have been created, however, only " + annotationCount++ + " were.");
153            }
154    
155            return annotationsToReturn;
156        }
157    
158        /**
159         * <p>
160         * This method searches for a String of text in a different String of text using regular expressions.
161         * <p>
162         * There is a known error with this method as entities with '' are sometimes not detected. This error may have something to do with ABNER's
163         * tokenization mechanism, as sometimes '' gets converted into ", and sometimes it does not.
164         * 
165         * @param subStr
166         * @param wholeStr
167         * @return
168         */
169        private Span findSpan(String subStr, String wholeStr) {
170    
171            /*
172             * One idiosyncracy of ABNER is that it converts '' to ", so we must do the same in "wholeStr". We add a space after the " so that the
173             * character offsets will remain consistent.
174             */
175            String processedWholeStr = wholeStr.replaceAll("''", "\" ");
176    
177            /* preprocess the string we are looking for by escaping any characters that need to be escaped in a regular expression */
178            subStr = GenericTextUtils.escapeCharacterForRegEx(subStr);
179    
180            String[] toks = subStr.split(" ");
181            String regex = "";
182            for (int i = 0; i < toks.length; i++) {
183                regex += toks[i];
184                if (i < toks.length - 1) {
185                    regex += "\\s*";
186                } else {
187                    regex += "";
188                }
189            }
190    
191            Pattern subStrPattern = Pattern.compile(regex);
192            Matcher subStrMatcher = subStrPattern.matcher(processedWholeStr);
193    
194            if (subStrMatcher.find(startLookingForMatchesAt)) {
195                Span span = null;
196                try {
197                    span = new Span(subStrMatcher.start(), subStrMatcher.end());
198                } catch (InvalidSpanException e) {
199                    e.printStackTrace();
200                }
201                startLookingForMatchesAt = subStrMatcher.end();
202                return span;
203            } else {
204                return null;
205            }
206    
207        }
208    
209        /**
210         * This method outputs an error message.
211         * 
212         * @param message
213         */
214        private void error(String message) {
215            System.err.println("ERROR -- Abner_Util: " + message);
216        }
217    
218    }
```
